# The effects of Sijunzi decoction-supplemented cat food on physiological parameters and gut microbiota in healthy adult cats

**DOI:** 10.3389/fvets.2025.1656675

**Published:** 2025-11-27

**Authors:** Wei Liu, Yinghao Chen, Mingyu Ma, Biao Liu, Jiayin Li, Yang Zhou, Junkun Yu, Jiahao Lin

**Affiliations:** 1 National Key Laboratory of Veterinary Public Health Security, College of Veterinary Medicine, China Agricultural University, Beijing, China; 2 Pet Food Department, Shenzhen Leiting Network Technology Co., Ltd., Shenzhen, China

**Keywords:** Sijunzi decoction, physiological parameters, gut microbiota, cat food, Chinese medicine

## Abstract

Cats have unique digestive characteristics that make them prone to gastrointestinal disorders, creating a growing demand for functional diets that support digestive and immune health. Traditional Chinese medicine (TCM) offers recognized advantages in gastrointestinal protection, yet its application in cat food remains rare. Among TCM formulas, Sijunzi Decoction (SJZD) has demonstrated benefits in enhancing appetite and protecting the intestine, yet its application in companion animal nutrition remains unexplored. In this study, we developed a kind of novel functional cat food that support intestinal health and immunity by incorporating the four constituent herbs of SJZD into the daily diet of healthy adult cats and assessed its effects on physiological parameters and gut microbiota in healthy adult cats. We displayed that a quality control system to ensure product consistency was successfully established. Further, we found that SJZD-supplemented cat food could promote digestion and nutrient absorption of cats, strengthens immune and antioxidant defenses of cats, and contribute to reducing the risks of obesity and inflammatory bowel disease of cats. In a word, this work provides both scientific evidence and a novel nutritional approach for improving feline health, while expanding the potential clinical applications of SJZD.

## 1 Introduction

In recent years, with rising living standards and faster-paced lifestyles, pets have become important sources of emotional support for families. Among them, cats gained particular popularity due to their small size, cleanliness, and relatively low maintenance requirements. However, the unique physiological features of cat digestive system pose nutritional challenges. Specifically, their digestive tract accounts for only 2.8–3.5% of body weight, while the stomach comprises about 60% of this system (1). Therefore, although cats possess strong digestive capacity, their relatively short intestines limit nutrient absorption and predispose them to gastrointestinal problems such as vomiting, diarrhea, and constipation. Unfortunately, these disorders can compromise immune function and overall health. Meanwhile, abundant clinical reports indicate that approximately 11.26% of cats visiting veterinary hospitals present with digestive diseases, ranking third among all causes of clinical visits (2, 3). So far, various therapeutic methods have been raised, including regular symptomatic treatments, supporting treatments and other adjuvant therapies. Among them, dietary strategies has huge advantages with simultaneous gastrointestinal health support and immunoregulation. Thus, cat owners increasingly demand diets that provide not only adequate nutrition and palatability but also additional health benefits (4).

Most of the cat food currently available on the market used beef, chicken, and other meats as the primary ingredients, resulting in fairly simple formulas (5). It was rare to find cat food that incorporated traditional Chinese medicine (TCM) formulations as the main ingredients. TCM had several advantages, including being natural, multifunctional, widely sourced, low in toxicity and side effects, and less likely to cause drug resistance. A TCM compound formula included multiple herbs and followed principles of combination and clinical experience, providing more comprehensive regulation and superior efficacy compared to single-ingredient medications (6). According to traditional Chinese veterinary medicine, the spleen was the postnatal foundation, responsible for transportation and transformation, ascending the nutrients, and containing blood (7). The spleen and stomach worked closely together, with their transformation functions providing essential nourishment to all organs and meridians. Strengthening the spleen was a practical application of TCM principles within the traditional Chinese veterinary medicine framework (8). It involved a holistic approach to maintaining balance and coordination among various body systems, aligning with modern medical concepts of digestive protection, immune regulation, and antioxidant functions.

Sijunzi Decoction (SJZD) was a traditional formula used to tonify qi of the spleen, consisting of *Codonopsis pilosula*, *Poria cocos*, *Atractylodes*, and *Glycyrrhiza glabra* (9). It was known for its ability to strengthen the spleen and supplement qi and was primarily used to treat qi deficiency of the spleen and stomach and poor appetite (10). *Codonopsis pilosula* was sweet in taste and neutral in nature, belonging to the spleen and lung meridians, and providing benefits such as tonifying qi of the spleen and stomach, tonifying the spleen, and benefiting the lung and nourishing blood and generating fluids (11). *Atractylodes* were bitter and sweet in taste, warm in nature, and belonged to the spleen and stomach meridians, helping to tonify the qi of the spleen, dry dampness, promote urination, and stop sweating (12). *Poria cocos* was sweet and bland in taste, neutral in nature, and belonged to the heart, lung, spleen, and kidney meridians, aiding in urination-promoting and dampness-draining, spleen-tonifying, and heart-calming (13). *Glycyrrhiza glabra* was sweet in taste and neutral in nature, belonging to the heart, lung, spleen, and stomach meridians, offering benefits such as tonifying qi of the spleen and stomach, clearing heat and removing the toxin, eliminating phlegm and relieving cough, relieving spasms and pain, and harmonizing the effects of other herbs (14).

Ma et al. have shown that the active component active component combination in SJZD can alleviate SDS-induced intestinal injury through the FAK/PI3K/Akt signaling pathway (15). Shi et al. indicated that SJZD polysaccharides can mitigate intestinal mucosal damage by affecting Ca^2+^-related regulators (16). Additionally, Xu et al. demonstrated that SJZD had the best immune-enhancing effects compared to Siwu Decoction and Bazhen Decoction (17). However, despite these well-recognized benefits, its application in companion animal nutrition has not been explored, and its potential role in promoting gastrointestinal health in cats remains unknown. Given that digestive disorders are among the most common clinical problems in cats, developing functional diets that support intestinal health and immunity has important practical value. Therefore, this study incorporated the four constituent herbs of SJZD into the daily diet of healthy adult cats and established a quality control system to ensure product consistency. We then evaluated physiological indicators and gut microbiota changes to systematically assess the effects of SJZD-supplemented cat food on digestion, immunity, and antioxidant function. By addressing a key gap between traditional knowledge and modern pet nutrition, this work provides both scientific evidence and a novel nutritional approach for improving feline health, while expanding the potential clinical applications of SJZD.

## 2 Materials and methods

### 2.1 TLC identification of *Codonopsis pilosula, Poria cocos, Atractylodes*, and *Glycyrrhiza glabra* in SJZD Chinese herbal ultrafine powder

The ultrafine powder of *Codonopsis pilosula, Poria cocos, Atractylodes*, and *Glycyrrhiza glabra*, and the corresponding control herbs were extracted 1 g each. They were mixed with 50 mL of anhydrous ethanol and sonicated for 10 min. After filtration, the filtrate was evaporated to dryness, and the residue was dissolved in 1 mL of methanol to prepare the test sample and control medicinal material solutions for subsequent thin-layer chromatography (TLC) identification.

### 2.2 TLC identification of *Atractylodes* and *Glycyrrhiza glabra* in cat food added with SJZD

Ten gram of cat food added with SJZD samples were finely ground and mixed with 10 g of diatomaceous earth. Then, 100 mL of 70% ethanol solution was added, followed by 30 min of sonication. After filtration, the filtrate was evaporated to dryness and the residue was dissolved in 5 mL of methanol to prepare the test sample solution. For negative control cat foods, 10 g of SJZD (without *Atractylodes*) and SJZD (without *Glycyrrhiza glabra*) were taken, and negative control solutions were prepared following the same method. Additionally, 10 g of blank cat food was taken to be prepared as a blank control solution using the same procedure. Subsequently, TLC identification was conducted on each solution.

### 2.3 Establishment of HPLC methodology for liquiritin detection in SJZD Chinese herbal ultrafine powder and cat food added with SJZD

The liquiritin reference standard, SJZD Chinese herbal ultrafine powder, Cat food added with SJZD test samples, and negative samples lacking liquiritin were prepared. Each of the above solutions was taken in 20 μL quantities. An Agilent XDB C18 chromatographic column (4.6 mm × 150 mm, 5 μm) was employed, with acetonitrile (A) - 0.05% phosphoric acid solution (B) used as the mobile phase for gradient elution. The chromatographic conditions for gradient elution were maintained: a flow rate of 1 mL/min, a column temperature of 25 °C, an injection volume of 20 μL, and detection at a wavelength of 220 nm. A series of standard solutions were prepared by precisely drawing volumes of the reference standard solution (162 μg·mL^−1^) into 5 mL volumetric flasks (3.5 mL, 2.5 mL, 1.5 mL, and 0.5 mL), and then making up the volume with methanol (chromatographic grade). After thorough mixing, standard solutions of various concentrations were obtained. Injection of these solutions was carried out according to the aforementioned chromatographic conditions for peak area determination. The standard curve of the reference standard was plotted using peak area as the dependent variable (Y) and mass concentration as the independent variable (X), and the regression equation and correlation coefficient were calculated. Following this, stability and repeatability assessments, as well as sample recovery tests, were conducted. Finally, the SJZD Chinese herbal ultrafine powder and cat food added with SJZD samples from the same batch were accurately weighed, and three test samples were prepared. These samples were sequentially injected according to the aforementioned chromatographic conditions to determine the content of liquiritin in the samples.

### 2.4 Effects of cat food added with SJZD on healthy adult cats

#### 2.4.1 Animal ethics statement

Eighteen healthy one-year-old cats were housed at the Shanchong Shuifu Pet Nutrition Research Center in Fangshan District, Beijing. The management conditions for each experimental group were identical, with a rearing space of 145 cm × 65 cm × 60 cm, a room temperature of (24 ± 2) °C, a relative humidity of (50 ± 5) %, and single-cage housing with free access to water. All cats had completed internal and external deworming and vaccination before the experiment. After a 4-week acclimation period, the cats were randomly divided into three groups: Control, TCM, and TCM-JN, with six cats in each group. There were no significant differences in initial body weight and body condition scores among the groups. The Control group was fed a normal diet, the TCM group was fed cat food with 1% SJZD additive, and the TCM-JN group was fed a basic diet along with SJZD capsules containing the same dosage as the TCM group. During the experiment, feeding was done at 09:00 and 17:00 daily, with free access to water. Food intake and body condition were monitored continuously over 24 weeks. At 4, 8, 12, 16, and 24 weeks, blood samples were collected from the hind leg saphenous veins of the experimental cats. Blood was collected using both EDTA anticoagulant and serum separation tubes. The serum tubes were left to stand at room temperature for 20–30 min and then centrifuged for 10 min (3,000 rpm, 24 °C) to collect the serum, which was stored at −20 °C. All animal experimental protocols used in this study were according to the guidelines for animal welfare and approved by the Ethics Committee of China Agricultural University and Experimental Animal Welfare (Ethical NO. AW13503202-2-3).

#### 2.4.2 Determination of BCS, FS, soft stool rate, and vomiting rate of cats in each group

The body condition score (BCS) and fecal score (FS) of each group of cats were monitored weekly. According to the 9-point BCS system, a score close to 5 was considered ideal (42). Based on the 5-point fecal scoring system, an FS score of 2–3 was ideal, while a score of ≥3.5 indicates soft stools (Table 1). The daily occurrences of soft stools and vomiting were recorded from 09:00 to 17:00. The soft stool rate for each cat was calculated using the formula: “Soft stool rate = (Number of soft stool days × Number of soft stool occurrences) / (Total number of cats × Total number of feeding days),” and the average soft stool rate for each group was determined. Similarly, the vomiting rate was calculated using the formula: “Vomiting rate = (Number of vomiting days × Number of vomiting occurrences) / (Total number of cats × Total number of feeding days),” and the average vomiting rate for each group was determined.

**Table 1:** 5-point fecal scoring system of the cat.

Grade	Grading basis
1	Hard, dry, small, and brittle lumps that crumble easily when pressed
1.5	The feces exhibited visible cracks, with an extremely dry exterior and a relatively dry interior, leaving no residue on the ground when picked up
2	Hard, well-formed, and dry feces, with only a small amount of residue left on the ground when picked up, maintaining its shape
2.5	Moist feces and slightly deform when picked up
3	Soft, well-formed, and moist feces, without cracks, and with a clear shape, deforming when picked up
3.5	Quite moist feces, with low viscosity, and unformed
4	Soft and unformed feces, with a high moisture content
4.5	Watery feces with low viscosity
5	Completely liquid feces

#### 2.4.3 Determination of IgG, IgM, cAMP/cGMP, T-AOC, SOD, MDA, MTL, GAS, and VIP in cat serum of each group

At every month of the overall test, the serum was collected from cats in each group for ELISA. Then, IgG, IgM, cAMP/cGMP, T-AOC, SOD, MDA, MTL, GAS, and VIP were determined according to kit instructions strictly.

#### 2.4.4 Determination of RBC, WBC, PLT, ALT, AST, TP, ALB, GLB, a/G, CREA, CHOL, and TBA in cat serum of each group

In the 4th, 12th, and 24th weeks, the serum was collected from cats in each group for hematological examination. Then, RBC, WBC, PLT, ALT, AST, TP, ALB, GLB, A/G, CREA, CHOL, and TBA were determined according to the Sysmex XN-1000 V automatic animal blood analyzer.

### 2.5 The analysis of 16S rRNA sequencing analysis of cat feces in each group

In the 4th, 12th, and 24th weeks, fecal samples were collected from the cats in sterilized tubes, with approximately 500 mg of feces collected from each cat, resulting in a total of 36 samples across the three groups. Total DNA extraction, quality control, and quantification of the fecal microbiome samples were first performed. Next, the bacterial 16S rDNA V3-V4 variable regions were targeted using universal bacterial primers for PCR amplification and library construction. The amplified PCR products were then quantified, recovered, and purified. Finally, the quantified amplification products from each group were pooled and sequenced for 16S bioinformatic analysis.

### 2.6 Statistics and analysis

SPSS 21.0 was used for statistical analysis in this study. The expression of data was mean ± standard deviation. *T*-test was used to compare the differences among groups after the detection of test data in the normal distribution *p < 0.05 meant a significant difference, **p < 0.05 meant a significant difference, and ***p < 0.001 meant a difference with extreme significance compared to the control group.

## 3 Results

### 3.1 Establishment and results of TLC identification and HPLC quantification of liquiritin in SJZD powder samples

**Figure 1:**
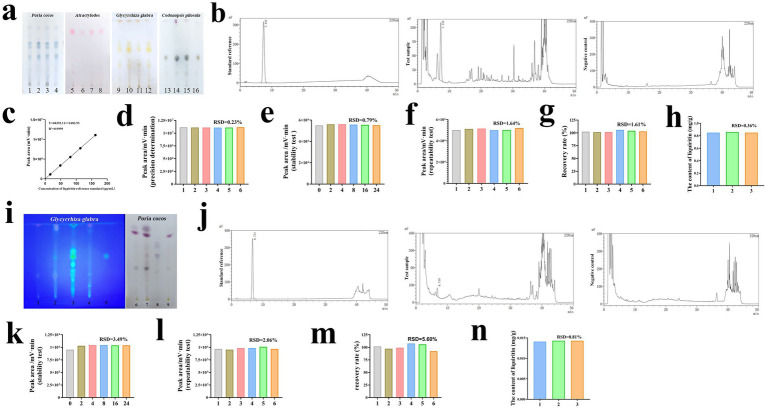
Quality control of SJZD samples and cat food added with SJZD. **(a)** The TCL results of *Codonopsis pilosula*, *Poria cocos*, *Atractylodes,* and *Glycyrrhiza glabra* in SJZD. 1 and 3 were test samples of *Poria cocos*, 2 and 4 were standard references of *Poria cocos*, 5 and 7 were test samples of *Atractylodes*, 6 and 8 were standard references of *Atractylodes*, 9 and 11 were test samples of *Glycyrrhiza glabra*, 10 and 12 were standard references of *Glycyrrhiza glabra*, 13 and 15 were test samples of *Codonopsis pilosula* and 14 and 16 were standard references of *Codonopsis pilosula*. **(b)** HPLC method specificity test results for determination of liquiritin in SJZD. **(c)** HPLC standard curves of liquirit in SJZD. **(d)** Results of precision determination of liquiritin in SJZD. **(e)** Results of stability test of liquiritin in SJZD. **(f)** Results of repeatability test of liquiritin in SJZD. **(g)** Results of recover rate of liquiritin in SJZD. **(h)** Results of the content of liquiritin in SJZD. **(i)** The TCL results of *Poria cocos*, and *Glycyrrhiza glabra* in cat food added with SJZD. 1 and 6 were the blank cat food samples, 2 and 7 were the samples of cat food added with SJZD, 3 were the standard references of *Glycyrrhiza glabra*, 4 were the samples of cat food added with SJZD lacking *Glycyrrhiza glabra*, 5 were standard references of liquiritin 8 were the standard references of *Poria cocos*, and 9 were the samples of cat food added with SJZD lacking *Poria cocos*. **(j)** HPLC method specificity test results for determination of liquiritin in cat food added with SJZD. **(k)** Results of stability test of liquiritin in cat food added with SJZD. **(l)** Results of repeatability test of liquiritin in cat food added with SJZD. **(m)** Results of recover rate of liquiritin in cat food added with SJZD. **(n)** Results of the content of liquiritin in cat food added with SJZD.

In the TLC identification of SJZD samples, the samples of *Codonopsis pilosula*, *Poria coco*s, *Atractylodes*, and *Glycyrrhiza glabra*, when compared with their respective reference materials, displayed spots of the same color at the corresponding positions. The separation between spots was distinct, indicating good specificity of the TLC method for these four herbs (Figure 1a). Specifically, samples and standards were as follows: 1 and 3 for Poria samples, 2 and 4 for Poria standards; 5 and 7 for Atractylodes samples, 6 and 8 for Atractylodes standards; 9 and 11 for Glycyrrhiza samples, 10 and 12 for Glycyrrhiza standards; 13 and 15 for Codonopsis samples, 14 and 16 for Codonopsis standards. For the HPLC analysis of liquiritin, the chromatographic peak for the liquiritin reference appeared at the corresponding retention time, with no interfering peaks in the negative control solution, indicating good specificity of the method (Figure 1b). The regression equation for liquiritin was Y = 68392.11X + 6183.93, with a linear range of 16.2 to 162 μg·mL^−1^ and an *R*^2^ value of 0.9999 (Figure 1c). The precision of the liquiritin peak area, expressed as relative standard deviation (RSD), was 0.23%, which is less than 2%, demonstrating good precision of the HPLC method (Figure 1d). The RSD of the liquiritin peak area was 0.79%, also less than 2%, indicating good stability of the test solution at room temperature for 24 h (Figure 1e). In six repeated extraction tests, the RSD of the liquiritin peak area was 1.64%, which was less than 2%, showing good repeatability of the method (Figure 1f). The average recovery rate of liquiritin was between 95 and 105%, with an RSD of 1.61%, which was less than 2%, indicating acceptable recovery rates (Figure 1g). In three samples, the liquiritin content was 0.855 ± 0.005 mg·g^−1^, with an RSD of 0.36%, demonstrating stable liquiritin content in the same batch of spleen-tonifying formula (Figure 1h). The control standard can be set at 85% of the average liquiritin content of this batch, meaning each gram of the product should contain no less than 0.73 mg of liquiritin. This HPLC method accurately determined the specificity, stability, precision, repeatability, and recovery rate of liquiritin in the spleen-tonifying formula.

### 3.2 Establishment and results of TLC identification and HPLC detection methods for liquiritin in SJZD-added cat food

The TLC identification method showed that the *Poria cocos* and *Glycyrrhiza glabra* cat food samples and their corresponding reference materials had identical colored spots with good separation, indicating high specificity (Figure 1i). The HPLC detection method also demonstrated high specificity, as there were no corresponding peaks at the liquiritin reference peak retention time in the negative control chromatogram, with no interference from other components and good separation (Figure 1j). The RSD of the liquiritin peak area was 3.49%, indicating good stability of the test solution at room temperature for 24 h (Figure 1k). The method showed good repeatability with an RSD of 2.06% (Figure 1l). The average recovery rate of liquiritin was between 95 and 105%, with an RSD of 5.60%, indicating good performance in the recovery test (Figure 1m). In three batches of samples, the average liquiritin content was 0.0142 ± 0.0001 mg·g^−1^, with an RSD of 0.81%, indicating stable content within the same batch of SJZD-added cat food samples (Figure 1n). A control standard can be set at 85% of this average content, meaning each gram of the product should contain at least 0.012 mg of liquiritin. This HPLC method can accurately determine the specificity, stability, precision, repeatability, and recovery rate of liquiritin in SJZD-added cat food.

### 3.3 Effects of SJZD-added cat food on BCS, FS, soft stool rate, and vomiting rate in cats

**Figure 2:**
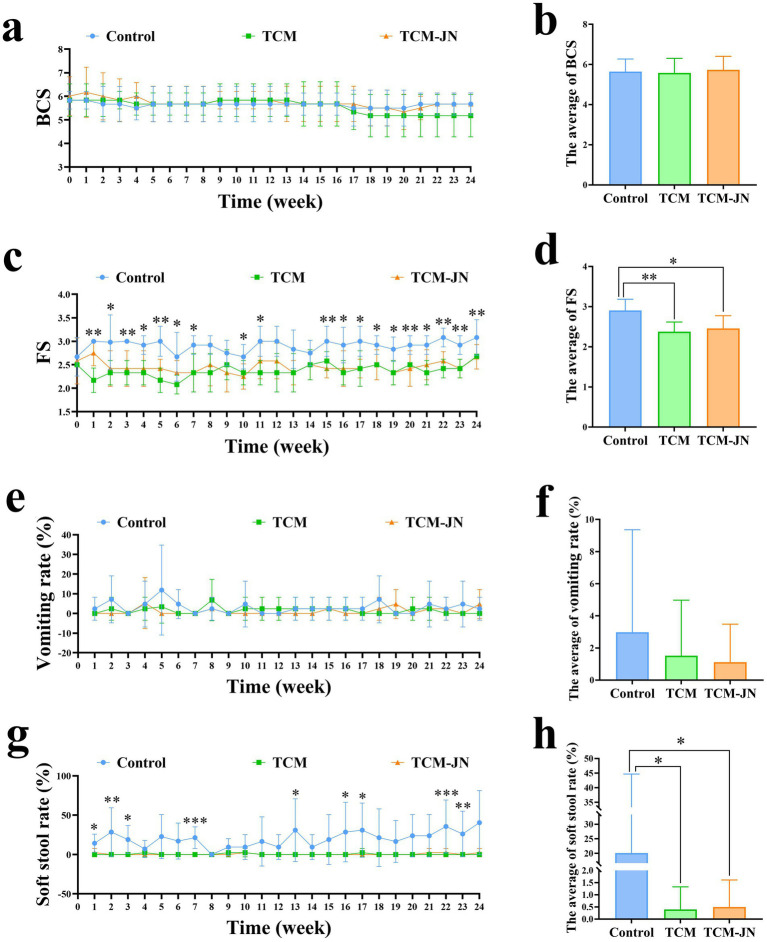
Results of BCS, FS, vomiting rate, and soft stool rate changes in cats throughout the feeding period. **(a)** The changing trend of BCS in cats throughout the feeding period. **(b)** The average change of BCS in cats throughout the feeding period. **(c)** The changing trend of FS in cats throughout the feeding period. **(d)** The average change of FS in cats throughout the feeding period. **(e)** The changing trend of vomiting rate in cats throughout the feeding period. **(f)** The average change of vomiting rate in cats throughout the feeding period. **(g)** The changing trend of soft stool rate in cats throughout the feeding period. **(h)** The average change of soft stool rate in cats throughout the feeding period.

During the 24-week trial, the average BCS of cats in the Control, TCM, and TCM-JN groups fluctuated but showed no significant differences (*p* > 0.05) (Figures 2a,b). However, significant differences in fecal scores were observed among the three groups in weeks 1, 2, 3, 4, 5, 6, 7, 10, 11, 15, 16, 17, 18, 19, 20, 21, 22, 23, and 24 (*p* < 0.05 or *p* < 0.01) (Figure 2c). The mean fecal scores for the Control, TCM, and TCM-JN groups were 2.91 ± 0.16, 2.37 ± 0.15, and 2.46 ± 0.24, respectively. Compared to the Control group, the TCM group showed a highly significant reduction in mean fecal score, while the TCM-JN group showed a significant reduction (*p* < 0.05) (Figure 2d). Significant differences in the soft stool rate were also found among the three groups in weeks 1, 2, 3, 7, 13, 16, 17, 22, and 23 (*p* < 0.05, *p* < 0.01, or *p* < 0.001) (Figure 2e). The mean soft stool rates for the Control, TCM, and TCM-JN groups were 20.49 ± 16.99%, 0.40 ± 0.72%, and 0.50 ± 0.70%, respectively. Both the TCM and TCM-JN groups showed a significantly lower mean soft stool rate compared to the Control group (p < 0.05) (Figure 2f). Throughout the trial, there were no significant differences in the vomiting rate among the three groups (*p* > 0.05) (Figures 2g,h).

### 3.4 Effects of SJZD on immune factors, antioxidant factors, and gastrointestinal hormones in cats

**Figure 3:**
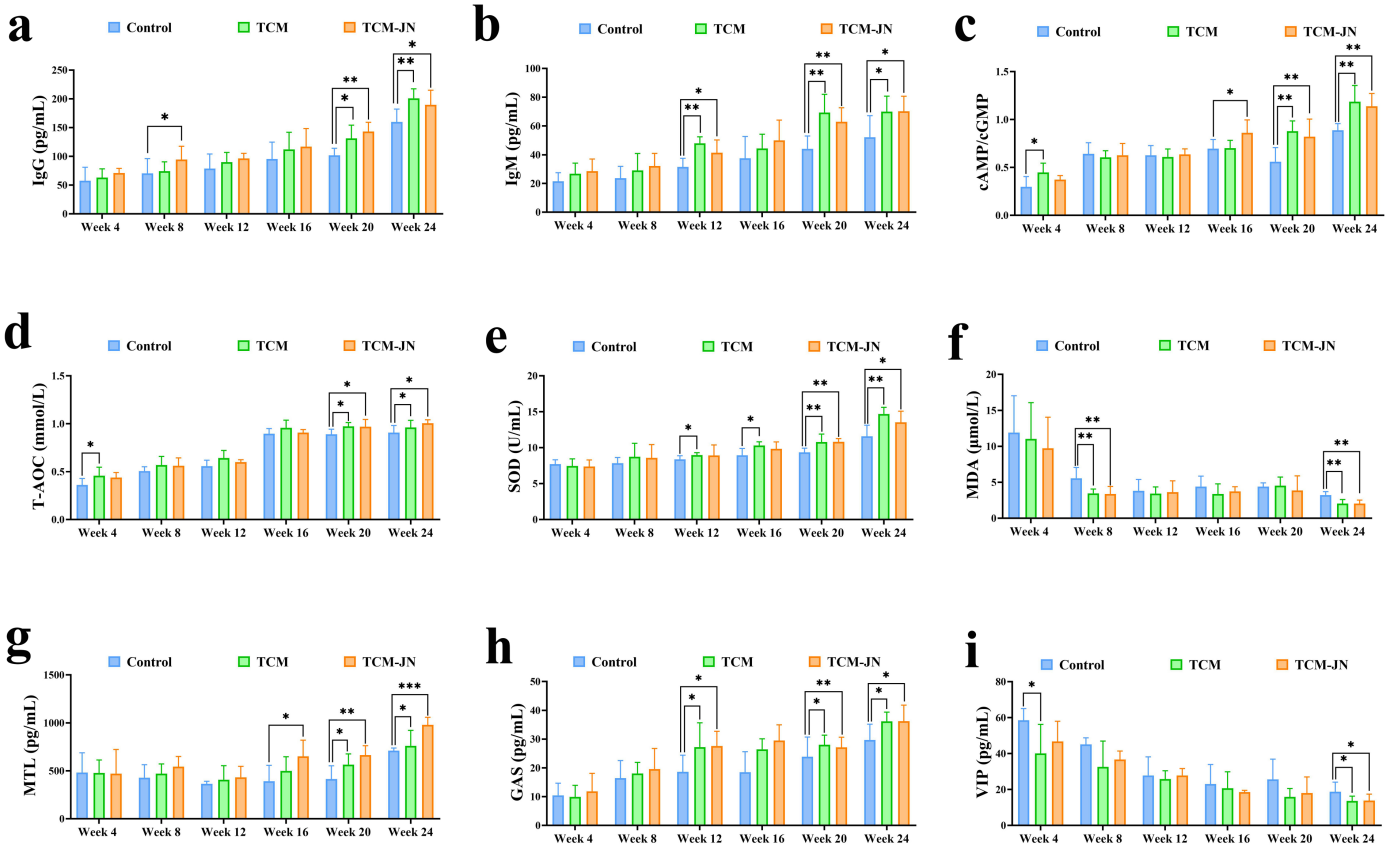
The levels of IgG, IgM, cAMP/cGMP, T-AOC, SOD, MDA, MTL, GAS, and VIP in serum from different groups of cats. **(a)** The level of IgG. **(b)** The level of IgM. **(c)** The level of cAMP/cGMP. **(d)** The level of T-AOC. **(e)** The level of SOD. **(f)** The level of MDA. **(g)** The level of MTL. **(h)** The level of GAS. **(i)** The level of VIP.

Compared to the control group, the level of IgG was significantly higher in the TCM group at the 20th and 24th weeks and in the TCM-JN group at the 8th, 20th, and 24th weeks in the serum of cats (*P* < 0.05 or *P* < 0.01) (Figure 3a). Compared to the control group, the level of IgM was significantly higher in the TCM and TCM-JN groups at the 12th, 20th, and 24th weeks in the serum of cats (*P* < 0.05 or *P* < 0.01) (Figure 3b). Compared to the control group, the level of cAMP/cGMP was significantly higher in the TCM group at the 4th, 20th, and 24th weeks and in the TCM-JN group at the 16th, 20th, and 24th weeks in the serum of cats (*P* < 0.05 or *P* < 0.01) (Figure 3c). Compared to the control group, the level of T-AOC was significantly higher in the TCM group at the 4th, 20th, and 24th weeks and in the TCM-JN group at the 20th, and 24th weeks in the serum of cats (*P* < 0.05) (Figure 3d). Compared to the control group, the level of SOD was significantly higher in the TCM group at the 12th, 16th, 20th, and 24th weeks and in the TCM-JN group at the 20th, and 24th weeks in the serum of cats (*P* < 0.05 or *P* < 0.01) (Figure 3e). Compared to the control group, the level of MDA was significantly lower in the TCM and TCM-JN groups at the 8th, and 24th weeks in the serum of cats (*P* < 0.01) (Figure 3f). Compared to the control group, the level of MTL was significantly higher in the TCM group at the 20th, and 24th weeks and in the TCM-JN group at the 16th, 20th, and 24th weeks in the serum of cats (*P* < 0.05, *P* < 0.01 or *P* < 0.001) (Figure 3g). Compared to the control group, the level of GAS was significantly higher in the TCM and TCM-JN groups at the 12th, 20th, and 24th weeks in the serum of cats (*P* < 0.05 or *P* < 0.01) (Figure 3h). Compared to the control group, the level of VIP was significantly lower in the TCM group at the 4th, and 24th weeks and in the TCM-JN group at the 24th week in the serum of cats (*P* < 0.05) (Figure 3i).

### 3.5 Effects of SJZD on blood routine and biochemical indexes of cats

**Figure 4:**
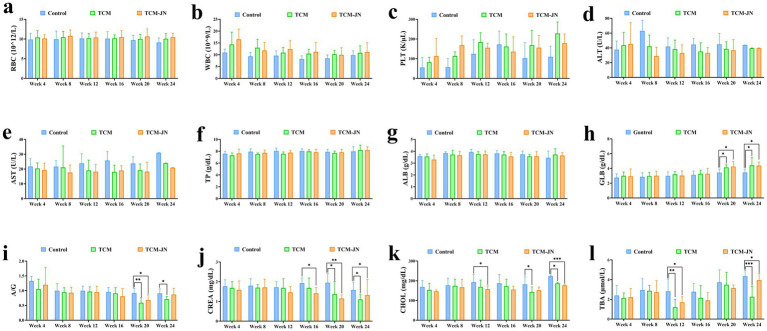
The levels of RBC, WBC, PLT, ALT, AST, TP, ALB, GLB, A/G, CREA, CHOL, and TBA in serum from different groups of cats. **(a)** The level of RBC. **(b)** The level of WBC. **(c)** The level of PLT. **(d)** The level of ALT. **(e)** The level of AST. **(f)** The level of TP. **(g)** The level of ALB. **(h)** The level of GLB. **(i)** The level of A/G. **(j)** The level of CREA. **(k)** The level of CHOL. **(l)** The level of TBA.

The levels of RBC, WBC, PLT, ALT, AST, TP, and ALB had no significant changes in serum from all different groups of cats (*P* > 0.05) (Figures 4a–g). Compared to the control group, the level of GLB was significantly higher in the TCM and TCM-JN groups at the 20th, and 24th weeks in the serum of cats (*P* < 0.05) (Figure 4h). Compared to the control group, the level of A/G was significantly lower in the TCM group at the 20th, and 24th weeks and in the TCM-JN group at the 24th week in the serum of cats (*P* < 0.05 or *P* < 0.01) (Figure 4i). Compared to the control group, the level of CREA was significantly lower in the TCM group at the 20th, and 24th weeks and in the TCM-JN group at the 16th, 20th, and 24th weeks in the serum of cats (*P* < 0.05 or *P* < 0.01) (Figure 4j). Compared to the control group, the level of CHOL was significantly lower in the TCM group at the 20th, and 24th weeks and in the TCM-JN group at the 12th and 24th weeks in the serum of cats (*P* < 0.05 or *P* < 0.001) (Figure 4k). Compared to the control group, the level of TBA was significantly lower in the TCM and TCM-JN groups at the 12th, and 24th weeks in the serum of cats (*P* < 0.05, *P* < 0.01, or *P* < 0.001) (Figure 4l).

### 3.6 Effects of SJZD on cat gut microbiota

**Figure 5:**
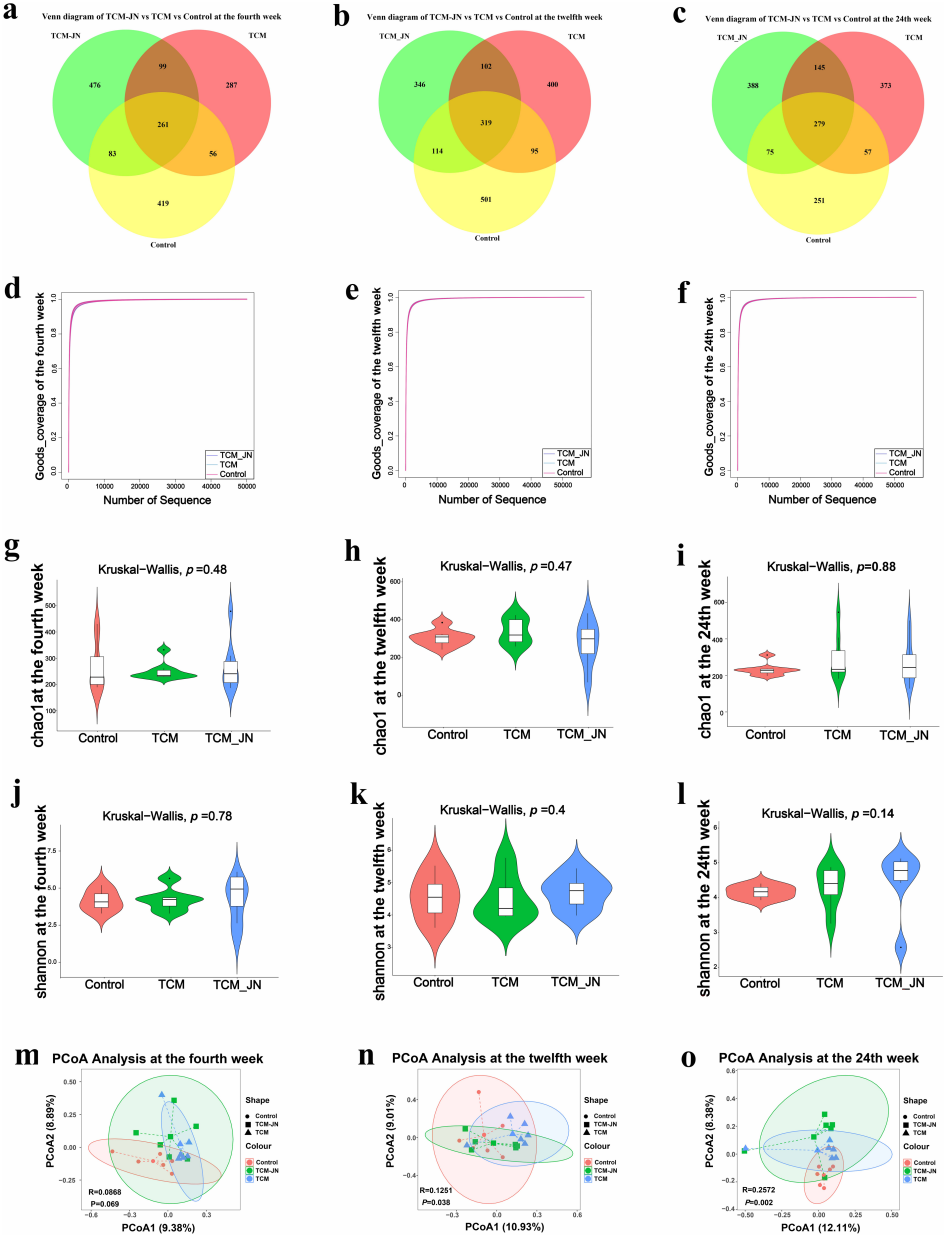
Effects of SJZD on cat gut microbiota. **(a)** The Venn diagram of three groups in the 4th week. **(b)** The Venn diagram of three groups in the 12th week. **(c)** The Venn diagram of three groups in the 24th week. **(d)** The Good-coverage of three groups in the 4th week. **(e)** The Good-coverage of three groups in the 12th week. **(f)** The Good-coverage of three groups in the 24th week. **(g)** The Chao 1 index of three groups in the 4th week. **(h)** The Chao 1 index of three groups in the 12th week. **(i)** The Chao 1 index of three groups in the 24th week. **(j)** The Shannon index of three groups in the 4th week. **(k)** The Shannon index of three groups in the 12th week. **(l)** The Shannon index of three groups in the 24th week. **(m)** The PCoA analysis of three groups in the 4th week. **(n)** The PCoA analysis of three groups in the 12th week. **(o)** The PCoA analysis of three groups in the 24th week.

In the 4th week, clustering of all samples revealed 1,504 ASVs, with the Control, TCM, and TCM-JN groups containing 819, 703, and 919 ASVs, respectively, among which 261 ASVs were common to all groups (Figure 5a). By the twelfth week, clustering yielded 1877 ASVs, with the Control, TCM, and TCM-JN groups containing 1,029, 916, and 881 ASVs, respectively, of which 319 were common (Figure 5b). At the twenty-fourth week, clustering resulted in 1668 ASVs, with the Control, TCM, and TCM-JN groups containing 662, 854, and 887 ASVs, respectively, of which 279 were common (Figure 5c).

α diversity analysis revealed good-coverage values exceeding 98% for all groups at weeks 4, 12, and 24, indicating sufficient sequencing depth to cover overall microbial diversity, allowing for data analysis (Figures 5d–f). Chao1 and Shannon indices showed a rising trend across weeks 4, 12, and 24 compared to the control group, albeit without significant changes, suggesting that SJZD-added cat food moderately increased gut microbiota richness (Figures 5g–l). β diversity analysis using Bray-Curtis measurements and PCoA revealed significant differences in gut microbiota structure among the Control, TCM, and TCM-JN groups at weeks 4, 12, and 24. Although SJZD supplementation did not alter species richness, it influenced the microbial structure of the gut microbiota. Notably, the gut microbiota structures of the TCM and TCM-JN groups were similar, indicating that SJZD-added cat food could stabilize the composition of gut microbiota (*p* < 0.05) (Figures 5m–o).

### 3.7 Effects of SJZD on cat gut microbiota at the phylum, family, and genus levels

**Figure 6:**
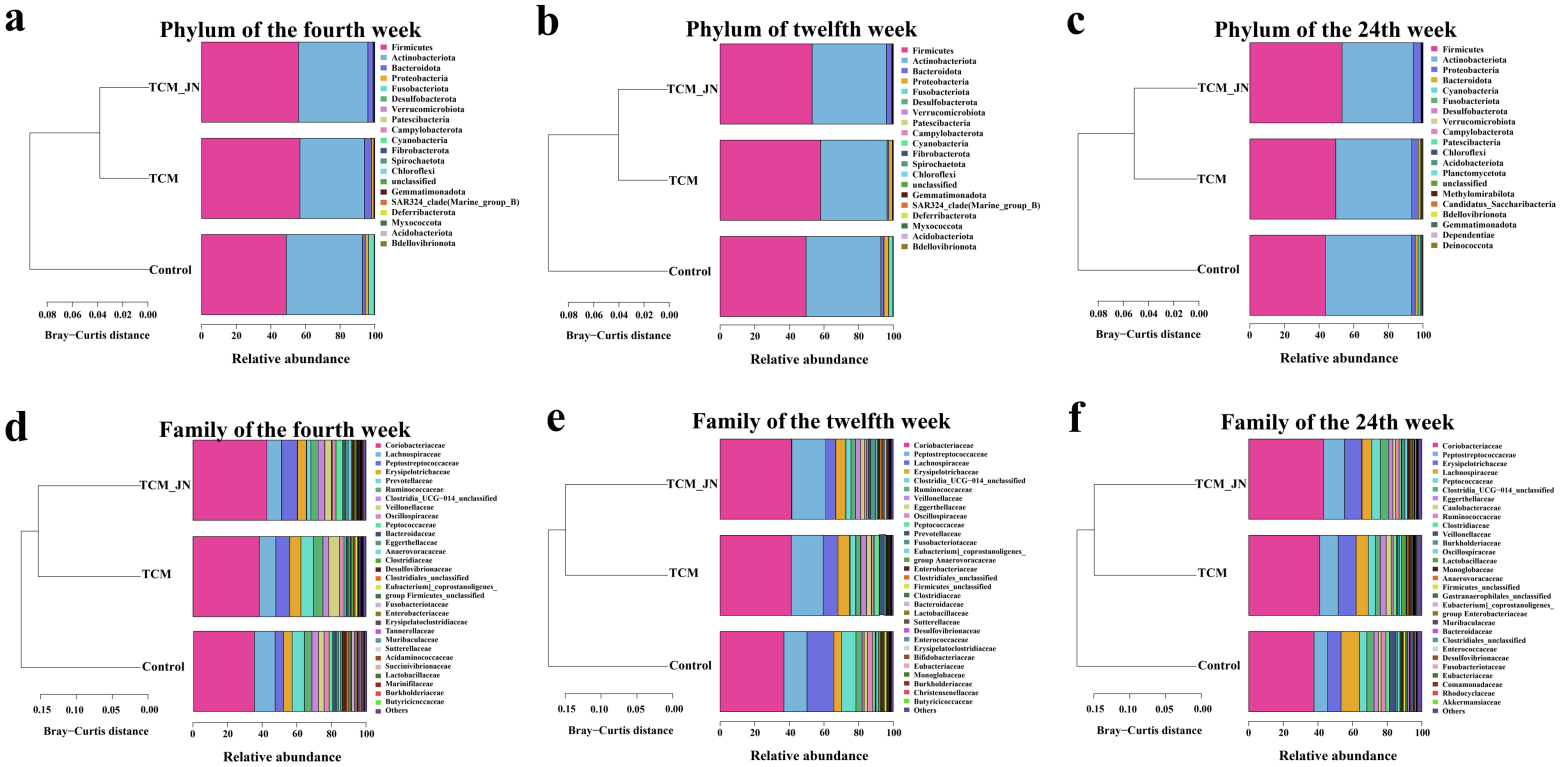
The results of SJZD on cat gut microbiota at the Phylum and Family levels. **(a)** The cluster on the Phylum level of three groups in the 4th week. **(b)** The cluster on the Phylum level of three groups in the 12th week. **(c)** The cluster on the Phylum level of three groups in the 24th week. **(d)** The cluster on the Family level of three groups in the 4th week. **(e)** The cluster on the Family level of three groups in the 12th week. **(f)** The cluster on the Family level of three groups in the 24th week.

To further investigate the specific impact of SJZD on the composition of the cat gut microbiota, differences in species composition at the phylum and family levels were analyzed. At the phylum level, the gut microbiota of cats in all groups at weeks 4, 12, and 24 were primarily composed of *Firmicutes*, *Actinobacteriota*, *Bacteroidota*, and *Proteobacteria*. SJZD addition mainly influenced the structure of *Firmicutes* and *Actinobacteriota*, increasing the relative abundance of *Firmicutes* and *Bacteroidota* while decreasing *Actinobacteriota* and *Proteobacteria* (Figures 6a–c). At the family level, the gut microbiota primarily consisted of *Coriobacteriaceae*, *Peptostreptococcaceae*, *Lachnospiraceae*, and *Erysipelotrichaceae*. SJZD addition mainly affected the structure of these four families, increasing the relative abundance of *Coriobacteriaceae*, *Peptostreptococcaceae*, and *Erysipelotrichaceae*, while decreasing *Lachnospiraceae* (Figures 6d–f).

**Figure 7:**
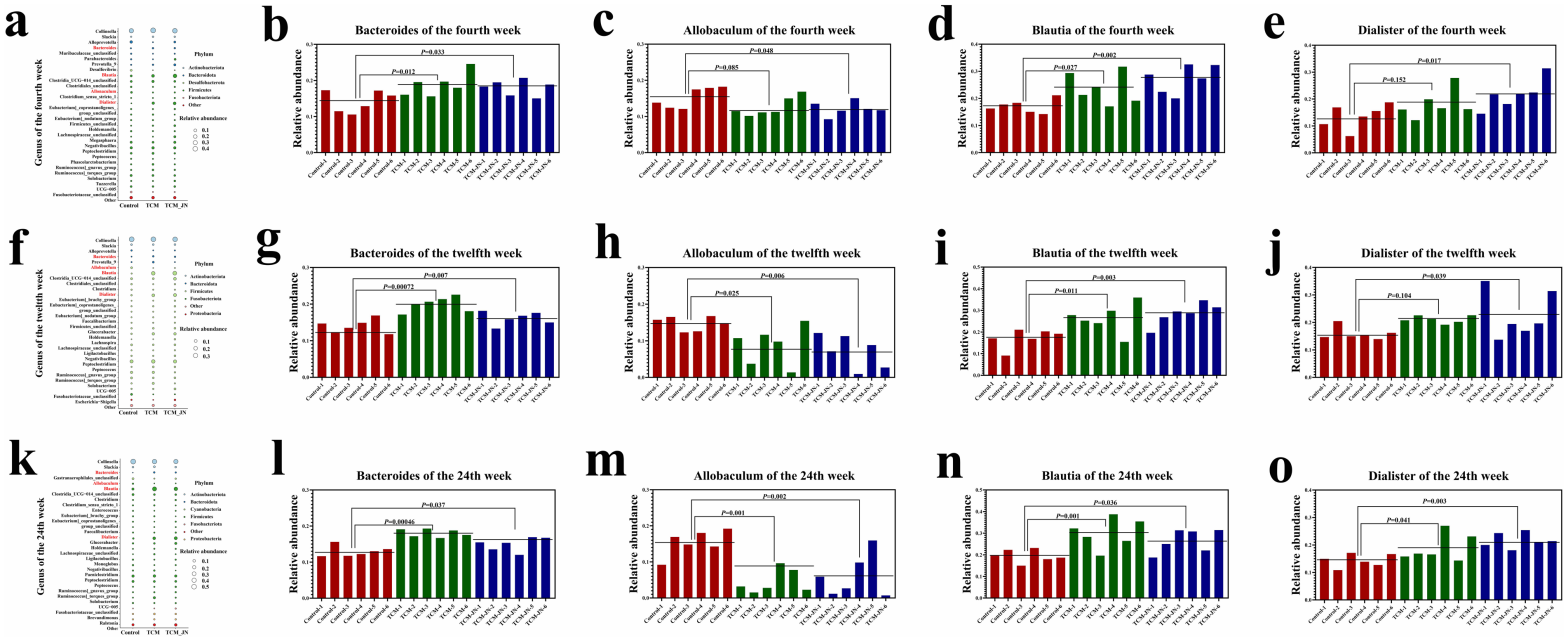
The results of SJZD on cat gut microbiota at the Genes levels. **(a)** The Bubble plot on the Genes level of three groups in the 4th week. **(b)** The relative *abundance* of *Bacteroides* among three groups in the 4th week. **(c)** The relative abundance of *Allobaculum* among three groups in the 4th week. **(d)** The relative abundance of *Blautia* among three groups in the 4th week. **(e)** The relative abundance of *Dialister* among three groups in the 4th week. **(f)** The Bubble plot on the Genes level of three groups in the 12th week. **(g)** The relative abundance of *Bacteroides* among three groups in the 12th week. **(h)** The relative abundance of *Allobaculum* among three groups in the 12th week. **(i)** The relative abundance of *Blautia* among three groups in the 12th week. **(j)** The relative abundance of *Dialister* among three groups in the 12th week. **(k)** The Bubble plot on the Genes level of three groups in the 24th week. **(l)** The relative abundance of *Bacteroides* among three groups in the 24th week. **(m)** The relative abundance of *Allobaculum* among three groups in the 24th week. **(n)** The relative abundance of *Blautia* among three groups in the 24th week. **(o)** The relative abundance of *Dialister* among three groups in the 24th week.

To determine the influence of SJZD on specific bacterial genera, we evaluated 32 significantly enriched genera. The results indicated that adding SJZD to cat food primarily increased the relative abundance of *Bacteroides*, *Blautia*, and *Dialister* while decreasing the relative abundance of *Allobaculum* (Figures 7a,f,k). Additionally, we observed changes in the relative abundance of these four specific genera across all samples between groups at weeks 4, 12, and 24 (*P* < 0.05, *P* < 0.01, or *P* < 0.001) (Figures 7b–e,g).

## 4 Discussion

We began by assessing the quality of TCM in SJZD-added cat food, ensuring control over the medicinal herbs’ quality at the source for the controllability of TCM cat food products. The test results indicated the stable and controllable quality of SJZD-added cat food products, complying with pharmacopeial standards. Subsequently, we investigated the effects of SJZD-added cat food on the BCS, FS, diarrhea rate, and vomiting rate of cats. Our findings revealed that SJZD-added cat food had no significant effect on regulating the vomiting rate of cats. However, after 18 weeks of feeding, cats in the SJZD-added cat food group had a lower BCS than those in the control group, indicating that cats consuming SJZD-added cat food were generally leaner than those in the control group, suggesting that SJZD-added cat food could control cat weight, thereby reducing the risk of obesity in cats. Furthermore, SJZD-added cat food significantly affected the FS and fecal scores of adult cats. The results of FS and fecal scores indicated that SJZD-added cat food could reduce the diarrhea rate of adult cats to a certain extent.

Subsequently, we further investigated the effects of adding SJZD to cat food on immune parameters in cat serum. Humoral immunity, mediated by B cells, was a crucial specific immune response and a major factor in disease resistance (18). The measurement of immunoglobulins in serum was the most common method for assessing humoral immune function (19). IgG and IgM, produced by activated B lymphocytes, were the primary immunoglobulins reflecting humoral immunity status. Studies have shown that *Codonopsis pilosula* increased the expression levels of IgG and IgM in aged mice, thereby enhancing immune function to delay aging (20). Lv et al. indicated that Poria polysaccharides elevated the serum levels of IgG and IgM in cyclophosphamide-induced immunosuppressed mice, exerting immunomodulatory effects (21). Our monthly measurements of serum IgG and IgM in adult cats revealed an increasing trend in the levels of these immunoglobulins with age. After adding SJZD to the cat food, the serum IgG and IgM levels at weeks 20 and 24 were significantly higher compared to the control group. This suggested that the SJZD used in this study enhanced the levels of immunoglobulins in cat serum, thereby modulating the immune function in adult cats.

Our results indicated that SJZD increased the ratio of cAMP to cGMP in cat serum. Cyclic adenosine monophosphate (cAMP) and cyclic guanosine monophosphate (cGMP) were crucial second messengers in biological organisms (22). They antagonized and constrained each other, jointly regulating normal cellular physiological functions (23). Due to their antagonistic and regulatory properties, cAMP was considered the “yang” in the yin-yang dynamic, while cGMP was considered the “yin” (24). *Codonopsis pilosula, Poria cocos, Atractylodes*, and *Glycyrrhiza glabra* can supplement spleen yin and exert yang-boosting effects, aligning with the observed increase in the cAMP/cGMP ratio. The spleen, the body’s largest immune organ, was closely linked to immune function. Gao et al. have shown that SJZD polysaccharides can enhance spleen cell proliferation and promote their immune functions (25). Thus, SJZD likely enhanced the physiological function of the spleen in cats, thereby amplifying its immunomodulatory capabilities and boosting the immune function in adult cats. Since the enhancement of immune function was a gradual process, there were no significant differences in serum IgG, IgM, and cAMP/cGMP levels between the SJZD group and the control group during the initial months of the study.

Our research results indicated that SJZD not only regulates the immune function of cats but also enhanced their antioxidant capacity. Total antioxidant capacity (T-AOC) represented the overall antioxidant level in cats, comprising various antioxidants and antioxidant enzymes (26). Superoxide dismutase (SOD) was the most important free radical scavenger in cats, playing a crucial role in protecting tissues from free radical damage (27). The activity level of SOD can be used to assess the body’s ability to neutralize free radicals. Polyunsaturated fatty acids were present in the biological membranes of cats. Reactive oxygen species (ROS) produced during metabolism can react with these fatty acids, leading to lipid peroxidation and the production of harmful by-products such as malondialdehyde (MDA) (28). The MDA content was an effective measure of the extent of free radical-induced tissue damage, helping to assess the impact of lipid peroxidation on tissue cells (29). Our results showed that SJZD increased the levels of T-AOC and SOD in the serum of adult cats while reducing MDA levels. This overall enhancement of the antioxidant capacity reduced the occurrence of lipid peroxidation. As a result, SJZD improved the ability of cat tissues to counteract free radicals and peroxides that caused tissue damage, preventing cellular structure impairment and DNA damage, and consequently reducing the risk of cancer.

The intestines were the body’s largest hormone-producing organ. Nutrient-sensing mechanisms in the specialized epithelial cells lining the gastrointestinal tract triggered the release of gut hormones. These hormones send important local and central feedback signals to regulate nutrient utilization and feeding behavior. Peptides distributed in the gastrointestinal tract and nervous system, such as brain-gut peptides, acted as messengers in this process. Motilin (MTL) was the primary regulator of gastrointestinal motility, while gastrin (GAS) was the main stimulant of gastric acid secretion. Both of them enhanced gastric motility, accelerated gastric emptying, and promoted small intestine movement. These brain-gut peptides served as biomarkers reflecting gastrointestinal function. Research has shown that irritable bowel syndrome with diarrhea (IBS-D) was associated with the release of vasoactive intestinal peptide (VIP) (30). High levels of VIP expression have been observed in the serum and colonic tissue of IBS-D rats. Other studies have found that rats with functional dyspepsia had lower levels of GAS and MTL in their serum, while VIP levels were significantly increased (31). Based on the analysis of fecal scores and the rate of soft stools, the reduction in the rate of soft stools in adult cats by SJZD may be related to its regulation of GAS and MTL levels.

Complete blood counts and biochemical parameters can quickly and accurately reflect the physiological and metabolic status of cats, serving as important indicators of overall health and organ function (32). Throughout the experiment, the blood counts and biochemical parameters of cats in all groups remained within normal ranges, suggesting that the addition of SJZD to cat food did not cause any adverse effects. However, our study found significant differences in globulin (GLB) and the albumin/globulin ratio (A/G) between cats fed SJZD-supplemented food and the control group. Based on the IgG and IgM test results, it was speculated that SJZD may increase the levels of IgG and IgM in cats, thereby raising serum GLB levels and lowering the A/G ratio to some extent. Additionally, the SJZD-supplemented diet significantly reduced serum creatinine (CREA) levels, which primarily reflected glomerular filtration function (33). This result suggested that SJZD may enhance the glomerular filtration function in cats, leading to a significant decrease in CREA levels within the normal range compared to the control group. Moreover, the study found that cats fed SJZD-supplemented food had significantly lower serum cholesterol (CHOL) and total bile acid (TBA) levels compared to the control group. Both CHOL and TBA were indicators of lipid metabolism, and their reduction implied that SJZD may improve lipid metabolic responses in cats, helping to prevent excessive obesity.

Increasing researchs indicated that gut microbiota played a crucial role in the body. Our study found that adding SJZD to cat food altered species richness to some extent and significantly affected the microbial structure of the gut microbiota in adult cats. At the phylum level, SJZD supplementation increased the relative abundance of *Firmicutes* and *Bacteroidota* while decreasing the relative abundance of *Actinobacteriota* and *Proteobacteria*. At the genus level, SJZD supplementation primarily increased the relative abundance of *Bacteroides*, *Blautia*, and *Dialister*, and decreased the relative abundance of *Allobaculum*. *Bacteroides* helped break down food, protected cats from pathogens, and provided nutrients for other gut microorganisms (34–36). Studies have shown that low levels of *Bacteroides* can lead to the onset and development of inflammatory bowel disease (IBD) (37). The increase in *Bacteroides* abundance in cats fed SJZD-supplemented food may enhance food digestion and absorption, protect against pathogenic microorganisms, and reduce the risk of IBD. This hypothesis was consistent with the observed serum levels of IgG, IgM, GLB, and A/G, suggesting that SJZD supplementation may modulate the immune system in adult cats.

Hosomi et al. have shown that *Blautia* can induce metabolic changes and exert anti-inflammatory effects in mice, reducing obesity and diabetes caused by a high-fat diet (38). Our 16S rRNA sequencing results revealed that the abundance of *Blautia* was significantly higher in the SJZD-supplemented group compared to the control group. We believed that SJZD supplementation may alter the abundance of *Blautia* species, thereby helping to activate metabolic processes and exert anti-inflammatory effects in cats. *Dialisters* were core members of the gut microbiota that can metabolize carbohydrates to produce succinate, acetate, propionate, and butyrate. Research has shown that *Dialisters* were associated with mood regulation and depression (39). Additionally, studies indicated that exercise can increase the abundance of *Dialisters* (40). Our study found that SJZD-supplemented cat food increased the abundance of *Dialisters*. Given that the metabolic products of *Dialisters* facilitated bidirectional communication between the microbiota-gut-brain axis, the impact of SJZD on *Dialisters* may have promising applications for alleviating depression in cats.

The abundance of *Allobaculum* was positively correlated with the expression of ANGPTL4. ANGPTL4 regulated lipid metabolism by inhibiting lipoprotein lipase (LPL), a key regulator of lipid metabolism and a mediator between the gut microbiota and fat deposition. Studies have shown that high-fat diets increased the expression levels of ANGPTL4 and the abundance of the *Allobaculum* genus in mice (41). Our experiment found that cats fed SJZD-supplemented food had reduced abundance of *Allobaculum*. We hypothesized that this reduction likely decreased ANGPTL4 expression levels, thereby increasing LPL activity, accelerating lipid metabolism, and reducing the risk of obesity. Based on BCS scores, after 18 weeks of feeding, cats in the SJZD-supplemented group had lower BCS scores compared to the control group, indicating that these cats were generally leaner. This finding corroborated the CHOL and TBA results, supporting our hypothesis, and suggested that SJZD-supplemented cat food mitigated the potential risks associated with obesity.

## 5 Conclusion

The quality of SJZD-supplemented cat food was stable and controllable, meeting the standards of the pharmacopeia. This cat food positively regulated cat weight, fecal scores, and the rate of soft stools. It enhanced serum immunoglobulin levels, boosted antioxidant capacity, improved glomerular filtration function, and elevated lipid metabolism levels. Additionally, it positively regulated the gut microbiota structure, aiding in the digestion and absorption of food, reducing the potential risks of obesity, and helping to protect against pathogenic microorganisms. It also exhibited anti-inflammatory effects and lowered the risk of IBD in cats. SJZD-supplemented cat food may provide a new nutritional supplement pathway to improve the overall health of adult cats.

## Data Availability

The datasets presented in this study can be found in online repositories. The names of the repository/repositories and accession number(s) can be found in the article/supplementary material.

## Glossary

BCS: Body condition scoring
FS: Fecal score
IgG: Immunoglobulin G
IgM: Immunoglobulin M
cAMP: Cyclic adenosine monophosphate
cGMP: Cyclic guanosine monophosphate
T-AOC: Total antioxidant capacity
SOD: Superoxide dismutase
MDA: Malondialdehyde
MTL: Motilin
GAS: Gastrin
VIP: Vasoactive intestinal peptide
RBC: Red blood cell
WBC: White blood cell
PLT: Platelet
ALT: Alanine transaminase
AST: Aspartate transaminase
TP: Total protein
ALB: Albumin
GLB: Globulin
A/G: Albumin/globulin
CREA: Creatinine
CHOL: Cholesterol
TBA: Total bile acid
